# Human CFTR deficient iPSC-macrophages reveal impaired functional and transcriptomic response upon *Pseudomonas aeruginosa* infection

**DOI:** 10.3389/fimmu.2024.1397886

**Published:** 2024-11-13

**Authors:** Claudio Rodriguez Gonzalez, Débora Basílio-Queirós, Anna-Lena Neehus, Sylvia Merkert, David Tschritter, Sinem Ünal, Jan Hegermann, Matthias Mörgelin, Jacinta Bustamante, Manuel Manfred Nietert, Ulrich Martin, Burkhard Tümmler, Antje Munder, Nico Lachmann

**Affiliations:** ^1^ Department of Pediatric Pneumology, Allergology and Neonatology, Hannover Medical School, Hannover, Germany; ^2^ Laboratory of Human Genetics of Infectious Diseases, Necker Branch, INSERM U1163, Necker Hospital for Sick Children, Paris, France; ^3^ Paris Cité University, Imagine Institute, Paris, France; ^4^ Division of Hematology/Oncology, Boston Children’s Hospital, Harvard Medical School, Boston, MA, United States; ^5^ Department of Pediatric Oncology, Dana-Farber Cancer Institute, Harvard Medical School, Boston, MA, United States; ^6^ Broad Institute of MIT and Harvard, Cambridge, MA, United States; ^7^ Leibniz Research Laboratories for Biotechnology and Artificial Organs (LEBAO), Department of Cardiothoracic, Transplantation and Vascular Surgery (HTTG), Hannover Medical School, Hannover, Germany; ^8^ Biomedical Research in Endstage and Obstructive Lung Disease Hannover (BREATH), Member of the German Center for Lung Research (DZL), Hannover Medical School, Hannover, Germany; ^9^ REBIRTH, Research Center for Translational and Regenerative Medicine, Hannover Medical School, Hannover, Germany; ^10^ Department of Medical Bioinformatics, University Medical Center Göttingen, Göttingen, Germany; ^11^ Research Core Unit Electron Microscopy, Institute of Functional and Applied Anatomy, Hannover Medical School, Hannover, Germany; ^12^ Colzyx AB, Lund, Sweden; ^13^ Study Center for Primary Immunodeficiencies, Necker Hospital for Sick Children, Assistance Publique-Hôpitaux de Paris AP-HP, Paris, France; ^14^ St. Giles Laboratory of Human Genetics of Infectious Diseases, Rockefeller Branch, The Rockefeller University, New York, NY, United States; ^15^ Cluster of Excellence RESIST (EXC 2155), Hannover Medical School, Hannover, Germany; ^16^ Fraunhofer Institute for Toxicology and Experimental Medicine, Hannover, Germany

**Keywords:** cystic fibrosis, macrophages, iPSC, infection, *Pseudomonas aeruginosa*, lung immunity

## Abstract

**Introduction:**

Cystic fibrosis (CF) is a hereditary autosomal recessive disease driven by deleterious variants of the CFTR gene, leading, among other symptoms, to increased lung infection susceptibility. Mucus accumulation in the CF lung is, as of yet, considered as one important factor contributing to its colonization by opportunistic pathogens such as *Pseudomonas aeruginosa*. However, in recent years evidence was provided that alveolar macrophages, which form the first line of defense against airborne pathogens, seem to be intrinsically defective with regard to bactericidal functionality in the CF lung. To assess the impact of CFTR deficiency in human macrophages only insufficient systems are available.

**Methods:**

To address this problem and to evaluate the role of CFTR in human macrophages, we successfully differentiated human induced pluripotent stem cells (iPSC) from a CF p.Phe508del homozygous individual and a healthy donor into primitive macrophages (iMac^ΔF508^ and iMac^WT^), respectively, and compared the bactericidal functionality in the relevant cell type.

**Results:**

iMac^ΔF508^ showed impaired *P. aeruginosa* clearance and intracellular killing capacity in comparison to iMac^WT^. Furthermore, iMac^ΔF508^ exhibited a less acidic lysosomal pH, and upon *P. aeruginosa* infection, there were signs of mitochondrial fragmentation and autophagosome formation together with a hyperinflammatory phenotype and deficient type I interferon response.

**Conclusion:**

In summary, we present a defective phenotype in iMac^ΔF508^ upon *P. aeruginosa* infection, which will constitute an ideal platform to further study the role of macrophages in the context of CF.

## Introduction

Cystic fibrosis (CF) is a monogenic autosomal recessive disease that results from deleterious variants of the *Cystic Fibrosis Transmembrane Conductance Regulator* (*CFTR*) gene. Globally, it affects more than 90,000 individuals and is most prevalent in individuals of European ancestry (1–3). CF is a multi-organ disease, specially affecting the lungs, pulmonary insufficiency being the leading cause of death of people with CF (pwCF), which accounts for around 80% of all fatalities (4). Although over 2,000 genetic variants of the *CFTR* gene have been identified (5), p.Phe508del (alternatively referred as ΔF508), a three base-pair in-frame deletion resulting in the absence of phenylalanine at position 508 of the CFTR amino acid chain, is the most common pathogenic CFTR variant in central Europe. Of note, the deletion of the single amino acid impairs the correct folding of the CFTR protein, resulting in its rapid degradation just after synthesis (6).

The *CFTR* gene encodes a protein that belongs to the ATP-binding cassette (ABC) transporter family. CFTR acts as a transmembrane channel, involved in the apical transport of chloride and bicarbonate ions across the epithelial cell membrane (7). In the CF lungs, CFTR impairment results in a perturbed ion and water homeostasis, causing dehydration and acidification of the airway surface liquid (8). This correlates with an abnormal aggregation of mucins, which leads to a very dense mucus layer, which is tightly adhered to the airway epithelium, impairing mucociliary clearance (9). Mucus accumulation facilitates infections with opportunistic pathogens, which in many cases persist over time as infections become chronic (10). The CF lung microbiota is an extremely variable and complex environment containing a wide range of commensals and opportunistic pathogens, including both fungi and bacteria (11–13). Here, *Pseudomonas aeruginosa* is a central player in CF lung deterioration due to its high prevalence and propensity to cause chronic colonization in the adult population of pwCF (14–16). The notoriously persistent nature of *P. aeruginosa* can be partially explained due to its facility to accumulate antimicrobial resistance and biofilm formation capacity (17, 18).

Macrophages are crucial effector cells of the innate immune system, performing diverse and vital roles ranging from the clearance of pathogens such as bacteria to tissue repair or modulation of inflammatory status (19, 20). Given the importance of alveolar macrophages in the defense against airborne pathogens (21), it is of relevance that multiple studies could indicate CF macrophages to have an impaired bactericidal capacity, hindering the cells’ capacity to efficiently resolve infections (22–26). In the context of the CF lung, suboptimal bactericidal functionality of alveolar macrophages sustains an inefficient pathogen clearance and waste management process, therefore creating a positive feedback loop on the already existing hyperinflammatory status of the CF lung, progressively damaging the tissue and ultimately leading to lung failure.

Despite the critical role of lung innate immunity in CF disease progression, research on the function of human macrophages in the CF lung is still scarce, especially in comparison to the expansive knowledge acquired on other cells involved in the CF pathogenesis, mainly epithelial cells. The lack of a reliable and standardized CF macrophage cell source is a major obstacle, and published research uses a variety of CF macrophages from heterogeneous sources. Most non-human studies have been performed in murine CF models, but also in ferret, rat and pig (27–30). However, all these animal disease models limit the extrapolation of study conclusions to pwCF. Therefore, the optimal cell source for CF macrophage research are pwCF; however, obtaining human biomaterials is not always feasible, and even in the best-case scenario, the unique CFTR variants, lifestyle, and medical history of each individual can introduce bias and reproducibility issues into published research.

To gain insights into the role of human CF macrophages in disease progression and develop a new tool for CF research we used iPSC-derived macrophages (iMac) as a robust biomaterial source. We differentiated human ΔF508 iPSC into macrophages (iMac^ΔF508^), and compared them with control cells, differentiated from a healthy iPSC line (iMac^WT^). iMac^ΔF508^ showed distinct functional, phenotypic and transcriptomic response upon *P. aeruginosa* infection, providing valuable insights into the intrinsic bactericidal defects of CF primitive macrophages.

## Materials and methods

### iPSC lines

The already established iPSC lines MHHi015-A (iPSC^WT^ (31) MHHi015-A; https://hpscreg.eu/cell-line/MHHi015-A, RRID: CVCL_ZX23), MHHi002-A (iPSC^ΔF508^ (32) https://hpscreg.eu/cell-line/MHHi002-A, RRID: CVCL_QX52) or MHHi005-A (also iPSC^ΔF508^, https://hpscreg.eu/cell-line/MHHi005-A, RRID: CVCL_VE60) were used in this project. Both lines were generated from peripheral blood isolates of donors using a lentiviral vector. The generation and use of iPSC lines was approved by the Local Ethics Committee at Hannover Medical School and informed consent was obtained from the donors. The patients/participants provided written informed consent to participate in this study. Further information regarding sex, age and ethnicity of the donors can be found online on the Human Pluripotent Stem Cell Registry, see links above (https://hpscreg.eu/).

### iPSC culture and differentiation

iPSC were either cultured in colonies over MEF feeder cells (CBA-310, Cell Biolabs) in the presence of embryonic stem cell (ESC) medium [KnockOut (KO) DMEM (Gibco) + 20% KO Serum Replacement (Gibco) + 1% L-Glutamine (200 mM, Gibco) + 1% P/S (10,000U/mL) + 1% NEAA (100x) + 0.2% 2- mercaptoethanol (50mM)] supplemented with 10ng/ml of freshly added fibroblast growth factor [bFGF, Gibco, reconstituted in 5mM Tris pH 7.6 and diluted in PBS + 0.1% bovine serum albumin (BSA) or as feeder-free monolayers in plates coated with Geltrex (ThermoFisher) and DMEM/F-12 Gibco) supplemented with Ascorbic Acid 2-Phosphate (64 mg/L, Sigma-Aldrich), Sodium Selenite (14 µg/L, Sigma-Aldrich), NaHCO_3_ (543 mg/L, Sigma-Aldrich), Insulin (20 mg/L, PAN), Human Recombinant Transferrin (10.7 mg/L Sigma-Aldrich), TGF-β (2ng/mL, Peprotech), bFGF (100 ng/mL, Peprotech), from now on described as E6 media. For feeder-based cultures, media was changed every 2-3 days. Once an iPSC line was expanded to at least 3x 6-well plates, the embryoid body (EB) formation process began. iPSC colonies were resuspended in ESC medium supplemented with 10µM Rock inhibitor and distributed in a 6-well suspension plate. The plate was placed on an orbital shaker at 85 rpm for 5-6 days, during which the medium was changed twice. For feeder-free cultures, 500x10^3^ cells were seeded per well of a suspension 6-well plate (GreinerBio) in the presence of E6 media supplemented with TGF-β (2ng/mL, Peprotech), bFGF (100 ng/mL, Peprotech), VEGF (50 ng/mL, Peprotech), BMP4 (50 ng/mL, Peprotech), SCF (20ng/mL, Peprotech) and Rock inhibitor (10µM, Tocris) for 24h under 70 rpm agitation. Next, media was replaced with E6 medium supplemented with VEGF (50 ng/mL, Peprotech), BMP4 (50 ng/mL, Peprotech), SCF (20ng/mL, Peprotech) and Rock inhibitor (10µM, Tocris) for 72h under 85 rpm agitation. On day 4, the media was replaced with E6 medium supplemented with VEGF (50 ng/mL, Peprotech), BMP4 (50 ng/mL, Peprotech), SCF (20ng/mL, Peprotech) Rock inhibitor (10µM, Tocris) and IL-3 (25ng/mL, Peprotech) for additional 72h under 85 rpm agitation. For both EBs formed under feeder-based or feeder-free culture, approximately 10-15 EBs were placed into a new well containing X-Vivo differentiation medium [X-Vivo 15 (Lonza) + 1% L-glutamine (200 mM) + 1% P/S (10,000 U/mL) + 0.1% 2-mercaptoethanol (50mM) + 25ng/ml hIL-3 (Peprotech) + 50ng/ml hMCSF (Peprotech); cytokines reconstituted in PBS + 0.1% BSA]. X-Vivo differentiation medium was changed every 7 days and from day 14 onwards, myeloid cell forming complexes originated and the attached EBs began to shed monocytes/macrophages in the media for the following 2-3 months, during which cells were harvested weekly from the supernatant. Harvested cells were terminally differentiated into iPSC-derived macrophages by incubating them for 5 days at a concentration of 1.5x10^6^ cells per well of a 6-well adherent plate (TPP) in Roswell Park Memorial Institute (RPMI) Differentiation Medium [RPMI 1640 (Gibco) + 10% FBS + 1% L-glutamine (200 mM) + 1% P/S (10,000 U/mL) + 50 ng/ml hM-CSF]. Mycoplasma absence was tested periodically along the differentiation process.

### CFTR gene sequencing

To sequence the portion of the genome responsible for the p.Phe508del pathogenic *CFTR* variant, genomic DNA was extracted from the terminally differentiated iMac using GenElute Mammalian Genomic DNA Miniprep Kit (Sigma) following manufacturer’s instructions. Two primers were designed to amplify a region of 1,102bp in the *CFTR* gene: CF_forward_: 5′- GTG CAT AGC AGA GTA CCT GAA ACA G-3′; CF_reverse_: 5′-TCA TAG TAA CAT ATT CCC TGC CCT A-3′. Afterwards, a PCR reaction was performed using Phusion High-Fidelity DNA polymerase (Thermo Scientific). Obtained amplicons were purified using QIAquick PCR Purification Kit (Quiagen) following manufacturer’s instructions. Finally, 125ng of purified PCR product was mixed with 25pmol of CF_forward_ primer in a 1.5ml tube and submitted to Lightrun sequencing services (Eurofins).

### iMac morphology analysis

In order to generate cytospin slides, 1-5x10^4^ terminally differentiated iMac were resuspended in 200μL of PBS and centrifuged into SuperFrost microscope slides (Thermo Scientific) by centrifugation (10’, 600g, RT) using a CytoSpin 4 (Thermo Scientific). The slides were then dried at RT and submerged for 5’ into May-Grünwald staining solution (Roth). After 3 washes using deionized water, slides were submerged for 20’ into Giemsa solution (Roth, 1:20). After another 3 washes the slides were dried overnight and fixed with ROTI Histokitt (Roth) mounting solution. Images of the cytospin slides and live iMac at the last day of terminal differentiation were taken using an Olympus IX71 microscope with the assistance of CellSens imaging software (Olympus).

### iMac surface marker expression

To characterize the surface marker expression of generated iMac, at least 1x10^5^ terminally differentiated cells were resuspended in 200µl of MACS buffer [PBS + 2% FBS + 2mM EDTA (Roth)]. Then, 1µl of human Fc-Blocking reagent (Human TruStain FcX, BioLegend) was added to the solution and incubated for 45’ at 4°C. Afterwards, the following antibodies were added to the respective cell solution, either for surface marker staining or isotype control: CD11b-FITC (ICRF44, 11-0118-41, eBioscience), CD14-PE (61D3, 12-0149-41, eBioscience), CD45-APC (HI30, 17-0459-42, eBioscience), CD86-PE (IT2.2, 12-0869-41, eBioscience), CD163-APC (GHI/61, 17-1639-41, eBioscience), CD206-PeCy7 (15-2, 321123, Biolegend), CD11b PE-Cy7 (ICRF44, 25-0118-42, eBioscience), CD11b-APC (ICRF44, 17-0118-41, eBioscience), CD14-FITC (61D3, 17-0149-42, eBioscience), CD45-eFluor™ 450 (HI30, 48-0459-42, eBioscience), CD15-Alexa Fluor 647 (MC-480, 125607, Biolegend), CD16-PE-Cy7 (CB16, 25-0168-42, eBioscience), CD1c-Brilliant Violet 605™ (L161, 331537, BioLegend), CD66b-FITC (G10F5, 305104, BioLegend), CD19-APC (HIB19, 17-0199-41, eBioscience), CD56-APC-Cy7 (5.1H11, 362511, BioLegend), CD282-PE (TL2.1, 12-9922-41, eBioscience), CD284-PE-Cy7 (HTA125, 25-9917-42, eBioscience), CD285-PE (85B152.5, MA5-16236, eBioscience), IgG1 κ iso-FITC (P3.6.2.8.1, 11-4714-41, eBioscience), IgG1 κ iso-PE (P3.6.2.8.1, 12-4714-81, eBioscience), IgG1 κ iso-APC (P3.6.2.8.1, 17-4714-81, eBioscience), IgG1 κ iso-PECy7 (P3.6.2.8.1, 25-4714-80, eBioscience), IgG2b k Iso-PE (eBMG2b, 12-4732-81, eBioscience), IgG1 κ iso-eFluor™ 450 (P3.6.2.8.1, 48-4714-80, eBioscience), IgG2a κ iso-PE-Cy7 (eBMa, 25-4724-81, eBioscience), IgM κ iso-FITC (MM-30, 401606, BioLegend), IgM κ iso-Alexa Fluor 647 (MM-30, 401618, BioLegend), IgG1 κ iso-Brilliant Violet 605™ (MOPC-21, 400161, BioLgened), IgG1 κ iso-APC-Cy7)MOPC-21, 400127, BioLegend), IgG2a κ iso-PE (eBMa, 12-4724-42, eBioscience). Surface marker expression was detected using a flow cytometer (CytoFLEX S, Beckman Coulter) and subsequently analyzed with the assistance of FlowJo 10 software. For compensation samples, OneComp eBead (Invitrogen) were used.

### *P. aeruginosa* culture and infection medium preparation

A culture of *P. aeruginosa* (reference strain PA14, DSM No.19882) was incubated overnight on 25 ml of LB media in an orbital shaker at 37°C and 120rpm. Afterwards, infection medium (RPMI 1640 + 10% FBS) was prepared at the appropriate multiplicity of infection (MOI) by measuring optical absorbance of the initial bacterial solution in a BioPhotometer plus (Eppendorf) and subsequent calculation of infection dose by applying the formula: 1 OD_600_ = 1.7x10^9^ colony forming units (CFU)/mL.

### Bacterial clearance assay

In order to assess the bacterial clearance capacity of the generated iMac, 1.5x10^5^ iMac were cultured in triplicate in RPMI 1640 medium without antibiotics 24 hours before the assay. On the following day, *P. aeruginosa* infection medium (RPMI 1640 + 10% FBS) was prepared at MOI 1 and 10. iMac cultures and corresponding wells without cells acting as bacterial growth controls (also in triplicates) were subsequently infected with the *P. aeruginosa* containing medium and after a centrifugation step (10’, 400g, RT), cells were incubated at 37°C in 5% CO_2_ and 92% humidity for 360’. Finally, CFU quantification of the supernatant from iMac cultures and respective bacterial growth controls was assessed using the drop plate method (33). In short, 7x 10-fold serial dilutions were prepared from the supernatant of the cultures and plated on LB agar plates. Finally, total CFU count was calculated and considering that 0% clearance capacity would be equal to the amount of CFU present in the bacterial controls and 100% clearance capacity would correspond to a hypothetical non-infected culture, the specific bacterial clearance capacity of each iMac line was determined.

### Bacterial killing assay

In order to assess the intracellular bacterial killing capacity of the generated iMac, 1.5x10^5^ iMac per well were cultured in 5 wells in RPMI 1640 medium without antibiotics 24 hours before the assay. On the following day, *P. aeruginosa* infection medium (RPMI 1640 + 10% FBS) was prepared at MOI 10. iMac were then infected and after a centrifugation step (10’, 400g, RT), incubated at 37°C in 5% CO_2_ and 92% air moisture for 60’. Afterwards, leftover bacteria in the supernatant were removed by 2x washing steps with PBS and 30’ incubation in RPMI 1640 + polymyxin B (100µg/ml, Fluka BioChemika) solution. After 2x washing steps with PBS to remove any residual antibiotic, the iMac from one well were lysed with a saponin (5mg/ml, SERVA) solution in PBS and the rest of the wells were left incubating with RPMI 1640 antibiotic-free medium. The CFU count of the lysed cell solution was subsequently determined by the aforementioned drop plate method. At different time points since the lysis of the first well (60’, 120’, 240’ and 360’), iMac were lysed, and viable intracellular bacteria quantified as CFU. Considering the number of CFU present in the earliest lysed well as the reference for 100% of viable intracellular bacteria, the % of alive bacteria on each subsequent time point was determined.

### Electron microscopy and quantification analysis

To corroborate the results of the killing assay, 4x10^6^ WT and CF iMac were cultured in cell culture petri dishes and subsequently exposed to *P. aeruginosa* following the bacterial killing assay. At time point 150’ post-infection (60’ after extracellular bacterial removal), instead of lysing iMac with saponin, a fixing solution was added [1.5% paraformaldehyde (PFA, Roth), 1.5% glutaraldehyde (Sigma), 150mM HEPES (Gibco), pH 7.35]. Cells were then stored at 4°C until they were detached using a cell scraper (VWR) and subsequently pelleted by centrifugation (5’, 300g). Cell pellets were then postfixed in 1% osmiumtetroxide for 2h, and then in 1% uranyl acetate overnight. After dehydration in acetone, pellets were embedded in EPON resin, cut into ultra-thin slices (50nm), and stained with 4% uranyl acetate. A FEI Morgagni transmission electron microscope operated at 80 kV accelerating voltage, with a Veleta (Olympus) side-mounted camera was used for non-immunolabeled samples. After a treatment with sodium metaperiodate (Merck) to unmask the antigens (34), the ultra-thin sections were immunolabeled with gold-labelled anti-Pseudomonas antibody (1:50 dilution; ab68538, Abcam) as previously described (35), using BSA (Aurion) as a blocking agent. Immunolabeled sections were examined in a Philips/FEI CM100 BioTwin transmission electron microscope operated at a 60 kV accelerating voltage and images were recorded with a Gatan Multiscan 791 charge-coupled device camera. For quantitative analysis, at least 53 immunolabeled intracellular *P. aeruginosa* residues in a minimum of 22 randomly selected iMac per cell type were evaluated in relation to their structural integrity and categorized as intact or digested bacteria. The image acquisition process and subsequent analysis was performed in a blinded manner. For the quantification of mitochondria organelles, micrographs containing mitochondria were analyzed using ImageJ software (36) to measure area and perimeter of organelles. Circularity of each mitochondria was calculated as (4πarea)/(perimeter^2^).

### Lysosensor green assay and bioinformatic analysis

In order to establish a comparative pH study of acidic organelles of both iMac lines, 1x10^5^ iMac were seeded in an 8- well uncoated µ-slide (Ibidi). 24 hours afterwards, iMac were washed with PBS and a 1μM solution of LysoSensor Green DND189 (Invitrogen) in Life Cell Imaging Solution (LCIS; 140mM NaCl + 2.5mM KCl + 1.8mM CaCl_2_ + 1mM MgCl_2_, + 20mM HEPES, pH 7.4) was added to the culture. After 15’ incubation at 37°C, cells were carefully washed twice with LCIS. Finally, images of stained cells were taken with Nikon Spinning Disk microscope using the 488nm laser at 20% power. At least 90 images from each iPSC line, generated in three consecutive differentiations, were analyzed using a bioinformatics program based on the open source KNIME platform designed to identify and measure dye intensity of cells and vacuoles within them. Selected parameters established a threshold of 1.5µm² maximum area for an event to be identified as a lysosome and an area between 200 and 500µm^2^ in order to be identified as a cell. The dye intensity of at least 5,200 acidic vacuoles from each cell line were measured and normalized to the background intensity of the cell to establish the fold-increase in dye intensity within the vacuoles, which is correlated to its pH (higher dye intensity=lower pH).

### iMac RNA isolation and sequencing

In order to analyze RNA expression of iMac during *P. aeruginosa* infection, duplicate samples of CF and WT iMac were subjected to the killing assay protocol. At time points 0, 60, 150 and 450’ post-infection, RNA was isolated using a RNeasy Micro Kit (Qiagen) following manufacturer’s instructions and subsequently preserved at -80°C. Once all samples passed a quality test to ensure the integrity and quantity of RNA, 220ng of total RNA per sample underwent a process of mRNA enrichment using NEBNext Poly(A) mRNA Magnetic Isolation Module (New England Biolabs). Afterwards, stranded cDNA libraries were generated using NEBNext Ultra II Directional RNA Library Prep Kit (New England Biolabs). Procedures were done downscaling 2/3 the initial volumes described in the user manual and one extra purification step was performed at the end of the protocol using Agencourt AMPure XP Beads (Beckman Coulter). Obtained cDNA libraries were barcoded using NEBNext Multiplex Oligos (New England Biolabs) and amplified with 9 cycles of PCR. To perform the sequencing run, barcoded libraries were pooled in equal molar amounts, each constituting 6.3% of the total flowcell capacity. After a denaturation step with NaOH and a subsequent dilution to 2.0pM, 1.3 ml of the pooled sample was loaded into an Illumina NextSeq 550 sequencer using a High Output Flowcell for single reads (Illumina). The processing of the raw sequencing data was performed using nfcore/rnased and Nextflow bioinformatics tools. The reference genome used to annotate the data was taken from GENCODE.org (Homo sapiens: GRCh38.p13; release 34). Data normalization and differential gene expression was analyzed with the bioinformatics tool Galaxy (version 20.05, RCU Genomics, Hannover Medical School, Germany) using default settings. Comparative analysis and figures were obtained with the bioinformatics tool Qlucore Omics explorer 3.7 filtering relative genes with P-values < 0.01 and fold change > 2. Gene set enrichment analysis was performed using the online platform Enrichr and GSEA software (37, 38). Given the exploratory nature of this study and following the GSEA website guidelines for exploratory datasets, a FDR cut-off of 25% and a P-value cut-off of 0,05 were used for this analysis, as a more stringent condition may lead to overlook potential significant results.

### Quantitative RT-PCR

Total RNA was reverse transcribed using the RevertAid First Strand cDNA synthesis kit (Thermo Fischer Scientific) following the manufactures instructions. Synthesized cDNA was used for qRT-PCR using a SYBR™ Green PCR Master Mix from Applied Biosystems (Schwerte, Germany) and the following QuantiTect primers assays from Qiagen (Hilden, Germany): *MX1* (QT00090895), *MX2* (QT00000581), *OAS1* (QT00099134, *IFIT3* (QT01002827 and *GAPDH* (QT00079247). Quantitative RT-PCR was performed using a 7500 Fast Real-Time PCR System from Applied Biosystems (Schwerte, Germany). Obtained Ct values were normalized to *GAPDH* Ct values. To confirm the expression of CFTR gene in terminally differentiated CF and WT iMacs, as well as comparing them to the expression levels of typical macrophage (THP-1) and epithelial cells (T84), RNA was extracted from all these four cell lines using RNeasy Mini Kit (Qiagen) following manufacturer’s instructions. Afterwards, cDNA was obtained from extracted RNA using the High-Capacity cDNA Reverse Transcription Kit with RNase Inhibitor (Applied Biosystems) following manufacturer’s instructions. Finally, qPCR reactions were performed using TaqMan-based detection (Applied Biosystems) with CFTR probe “Hs00357004_m1” and GAPDH probe “Hs02786624_g1” as housekeeping gene. Obtained CFTR Ct values were then normalized to the GAPDH Ct values and using the 2^−ΔΔCt^ method (39), the fold-change CFTR expression of iMacs was compared to epithelial and macrophage reference cell lines.

### Detection of p47phox phosphorylation

To detect phosphorylation status of p47phox, 0.5x10^5^ terminally differentiated iMacs of each line were seeded per well in 12-well plates. 24 h later, cells were exposed to either a PMA solution (40ng/ml) for 15’ at 37°C, or a *P. aeruginosa* infection (PA14, MOI 10) at 37°C for 15’ or 30’. After the respective incubation times, cells were lysed using 200µl of RIPA buffer (Sigma), supplemented with 10% DTT (100mM), protease inhibitor (complete Protease Inhibitor Cocktail; Sigma) and phosphatase inhibitor (Halt Phosphatase Inhibitor Cocktail; Thermo Scientific). Cell lysates were then incubated on ice for 25’ and centrifuged for 20’ at 13,000g and 4°C. Finally, the supernatant was stored at -80°C. To perform the western blot, 20µg of protein from each experimental condition were resolved by electrophoresis in a 10% Criterion TGX precast gel (Bio-Rad) and ran for 45’ at 45mA. Afterwards, the obtained protein bands were transferred onto a nitrocellulose membrane (Bio-Rad) using a Transblot turbo system (Bio-Rad). The membrane was then blocked with a solution of 3% BSA in PBS-T (PBS+0.1% Tween20) and subsequently incubated overnight at 4°C with the primary antibodies: p47phox 1:4000 (07-001, Merck), and p-p47phox 1:1000 (S328, gift from Jamel El-Benna). Finally, proteins were detected by chemiluminescence after an incubation step with a goat anti-rabbit IgG (H+L)-HRP secondary antibody (Bio-Rad). Equal loading was assessed by using an anti-vinculin-HRP antibody (1:3000 diluted, # sc-73614, Santa Cruz Biotechnologies). For densitometric analysis, band intensity of p47phox and p-p47phox were measured and normalized to the background of each lane. Afterwards, the ratio p-p47phox/p47phox was calculated and normalized to the non-stimulated condition.

### Statistical analysis and figure creation

Result visualization and statistical analysis were performed using GraphPad Prism software (Version 9). Graph bars represent mean ± standard deviation unless otherwise stated. Applied statistical tests are specified in each figure legend. Statistical significance was measured in P-value and indicated with asterisks (*p < 0.05, **p < 0.01, ***p < 0.001 and ****p < 0.0001). Schematic figures were created with BioRender.com.

## Results

### Successful generation of macrophages from healthy and CF iPSC lines

**Figure 1:**
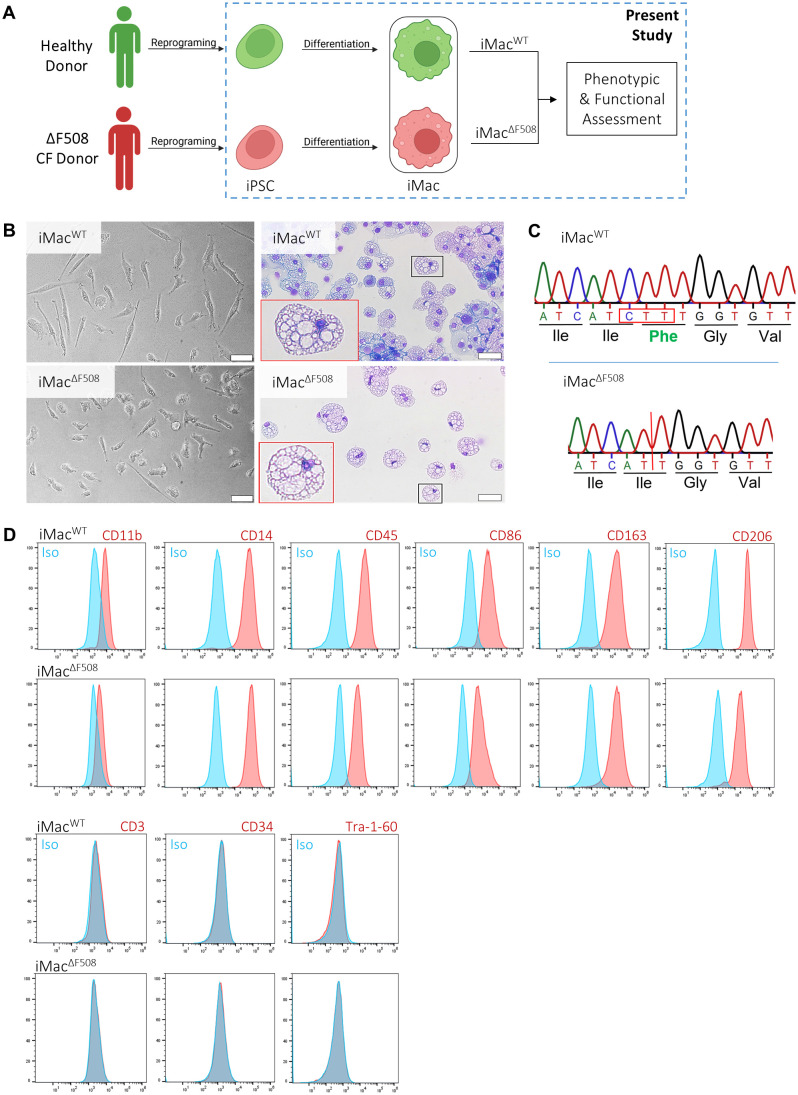
Phenotypic, morphological and genetic characterization of iPSC-derived macrophages from healthy and patient donors. **(A)** Scheme depicting the experimental pipeline, from cell donors to the phenotypic and functional assessment of both lines of iPSC-derived macrophages (iMac). **(B)** Representative bright field microscopy of iMac in culture (left column) and fixed on cytospin slides (right column). Scale bar: 50µm. **(C)** Sequencing of a portion of the *CFTR* gene with detection of homozygous triple base deletion of variant p.Phe508del can be seen in iMac^ΔF508^. **(D)** Phenotypic characterization of iMac surface marker expression (red) and isotype controls (blue). Both iMac lines were characterized for the expression of the following typical macrophage surface markers:CD11b, CD14, CD45, CD86, CD163 and CD206. Stem and T-cell markers were incorporated in the immunophenotypic panel in order to exclude possible cell contaminants (Tra-1-60, CD34 and CD3).

This study used previously published iPSC lines obtained by reprogramming primary cells form a healthy donor and a CF p.Phe508del homozygous individual (31, 32). Both iPSC lines (iPSC^WT^ and iPSC ^ΔF508^) underwent an already established protocol (40) aimed to generate iPSC derived macrophages (iMac^WT^ and iMac^ΔF508^, respectively). Subsequently, phenotypic and functional assessments of both iMac lines were performed (**Figure 1A**).

Morphology of cells was assessed by bright field microscopy. Therefore, images of terminally differentiated iMac were taken either directly in culture or spotted on a cytospin slide and stained with May–Grünwald–Giemsa. At the end of the differentiation process, both iMac lines showed typical macrophage size and morphology (**Figure 1B**).

The portion of the genome associated with the p.Phe508del mutation in the *CFTR* gene of the generated iMac was sequenced. iMac^ΔF508^ presented the expected genotype of a CF individual homozygous for p.Phe508del, a three base pair deletion (CTT) resulting in the absence of a phenylalanine residue in the CFTR amino acid chain (**Figure 1C**). Furthermore, also as expected, CFTR mRNA expression of iMac^WT^ and iMac^ΔF508^ was comparable to a macrophage cell line (THP-1), but hundreds of times lower when compared to an epithelial cell line (T84) and slightly higher in iMac^WT^ when compared to iMac^ΔF508^ (**Supplementary Figure 1A**).

Moreover, terminally differentiated iMac were characterized by flow cytometry analysis. Similar amounts of the following typical macrophage surface marker expression were detected in both lines: CD11b, CD14, CD45, CD86, CD163, CD206 as well as TLR-2, TLR-4 and TLR-5. Furthermore, the absence of cell marker expression associated with stem cells (Tra-1-60), hematopoietic stem cells (CD34), T-cells (CD3), B cells (CD19), NK cells (CD56), dendritic cells (CD1c), granulocytes and monocytes (CD15 and CD66b), discarded a potential contamination with other cell types from both lymphoid and myeloid lineages and non-differentiated pluripotent cells, confirming a successful and comparable iMac differentiation process for both iPSC lines (**Figure 1D**; **Supplementary Figure 1B**).

### CF iMac exhibit an impaired bactericidal capacity against *P. aeruginosa*

**Figure 2:**
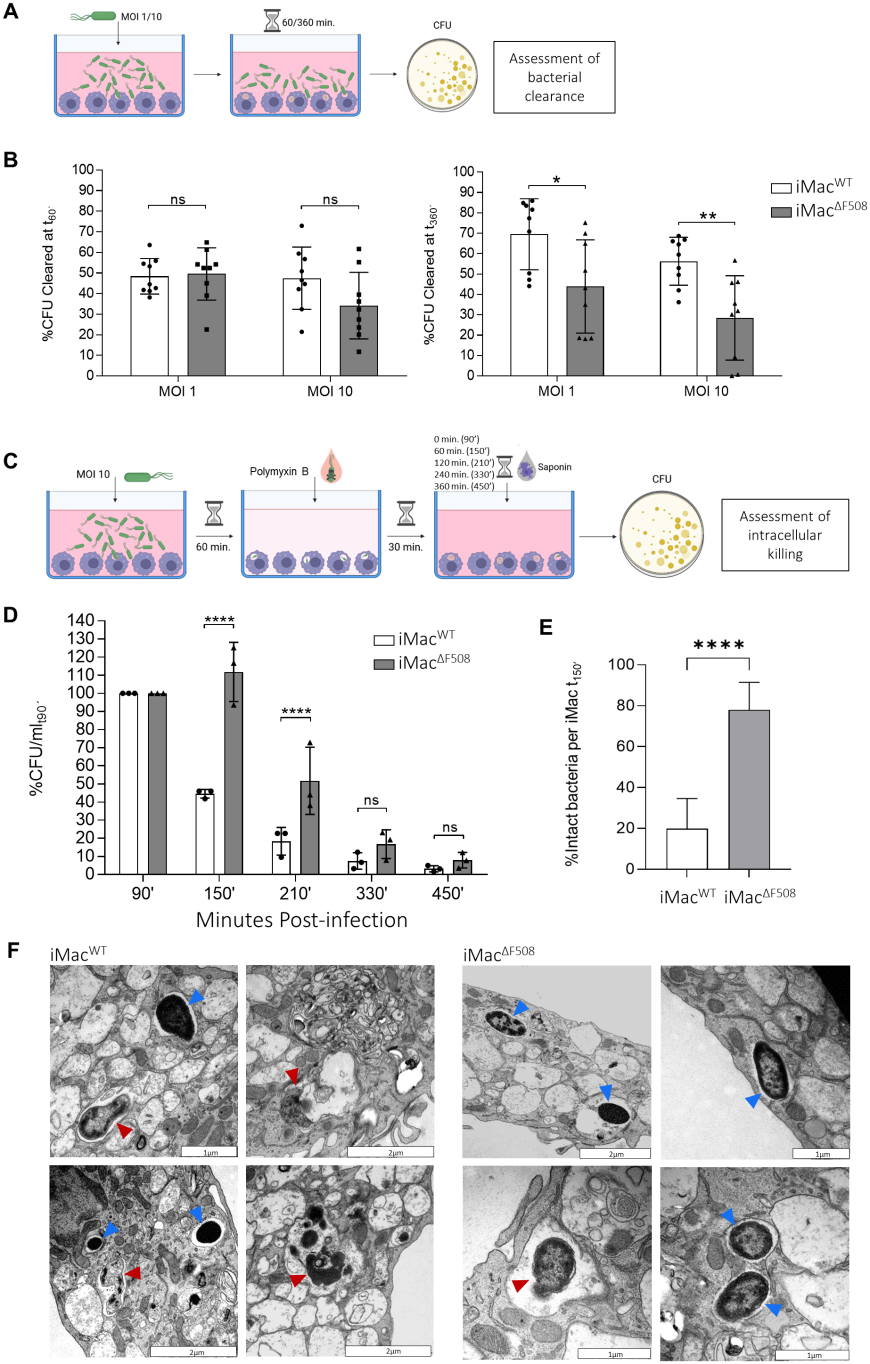
Assessment of iMacs^WT^ and iMacs ^ΔF508^ clearance capacity of *P. aeruginosa*. **(A)** Scheme describing the functional testing of iMac general bactericidal clearance capacity. **(B)** Bar graphs depict the bacterial clearance by iMac^WT^ (white bars) and iMac ^ΔF508^ (grey bars) at 60’ (left) and 360’ (right) post-infection at MOI 1 and 10. N=9 per MOI and iMac line analyzed. 2-way ANOVA and Tukey’s multiple comparison test. **(C)** Scheme describing the functional testing of iMac intracellular bacterial killing capacity. **(D)** Quantification of **i**ntracellular killing of *P. aeruginosa* by iMac^WT^ and iMac^ΔF508^ at time points 90’, 150’, 210’, 330’ and 450’post-infection. N=3 per time point and macrophage line analyzed. 2-way ANOVA and Tukey’s multiple comparison test. **(E)** Structural integrity analysis of internalized *P. aeruginosa* at 150’ post-infection. Bars represent mean ± 95% confidence interval of percentage of intact bacteria inside iMac^WT^ and iMac^ΔF508^. Twenty-two and 24 cells were analysed for iMac^WT^ and iMac^ΔF508^, respectively (N=22 and N=24, Mann-Whitney-U test). **(F)** Representative micrographs of internalized bacteria on both iMac lines at 150’ post-infection. Red arrows highlight degraded internalized bacteria, while blue arrows highlight intact internalized bacteria. Scale bar: 1 or 2µm. P-value indicated with asterisks (*p < 0.05, **p < 0.01 and ****p < 0.0001).

In order to assess the general bacterial clearance capacity of generated iMac^WT^ and iMac^ΔF508^, they were first exposed to *P. aeruginosa* PA14 at MOI 1 or 10. After a co-culture of 60’ or 360’, CFU in the supernatant were assessed (**Figure 2A**). CFU were compared with a bacterial growth control without the presence of macrophages so that the percentage of successfully cleared CFU of each respective condition could be calculated. When the co-culture time was limited to 60’, no significant functional difference was detected between iMac^WT^ and iMac^ΔF508^. When iMac were infected at MOI 1 for 60’, both lines cleared approximately 50% of CFU, however, when exposed to higher infection doses (MOI 10), iMac^ΔF508^ tended to exhibit a reduced bacterial clearance (approx. 35% clearance vs. 50% done by WT cells). More striking results were obtained when the co-culture was prolonged to 360’. In those conditions, iMac^WT^ were significantly more efficient at clearing bacteria in comparison to iMac^ΔF508^ at both tested MOIs (1 and 10). At MOI 1, iMac^WT^ cleared approximately 70% of extracellular bacteria while iMac^ΔF508^ only reached approximately 45%. Furthermore, at higher infection dose (MOI 10), iMac^WT^ cleared approximately 55%, while iMac^ΔF508^ only cleared 30% of extracellular bacteria (**Figure 2B**).

To further understand the observed decreased general bacterial clearance functionality of iMac^ΔF508^, the intracellular bacterial killing capacity of both cells was assessed. In short, iMac were first infected with MOI 10 of *P. aeruginosa*. After 60’ during which cells could freely phagocytose extracellular bacteria, the culture was treated with antibiotics in order to remove non-phagocytosed bacteria, so cells could be lysed at different time points and intracellular viable CFU could be counted (**Figure 2C**). The result of this assay confirmed the impairment of iMac^ΔF508^ regarding their intracellular bacterial killing capacity. This defect was especially obvious after the first hour of culture in a *P. aeruginosa* free medium (time point 150’). While iMac^WT^ efficiently eradicated approximately 50% of their intracellular bacteria, iMac^ΔF508^ still kept an amount of viable *P. aeruginosa* comparable to that of the previous time point (90’). The number of internalized bacteria of iMac^ΔF508^ decreased in following time points but did not reach a level similar to the iMac^WT^ until 330’ post-infection (**Figure 2D**).

To further complement these findings, internalized *P. aeruginosa* residues at time point 150’ post-infection were analysed regarding their structural integrity. In correlation with the previous result, the amount of structurally intact, and therefore potentially viable bacteria, was significantly higher within iMac^ΔF508^ in comparison to iMac^WT^ (**Figure 2E**), as evidenced by the analysis of iMac^WT^ and iMac^ΔF508^ micrographs (**Figure 2F**).

### CF iMac exhibit distinct lysosomal, mitochondrial and phagosomal characteristics

**Figure 3:**
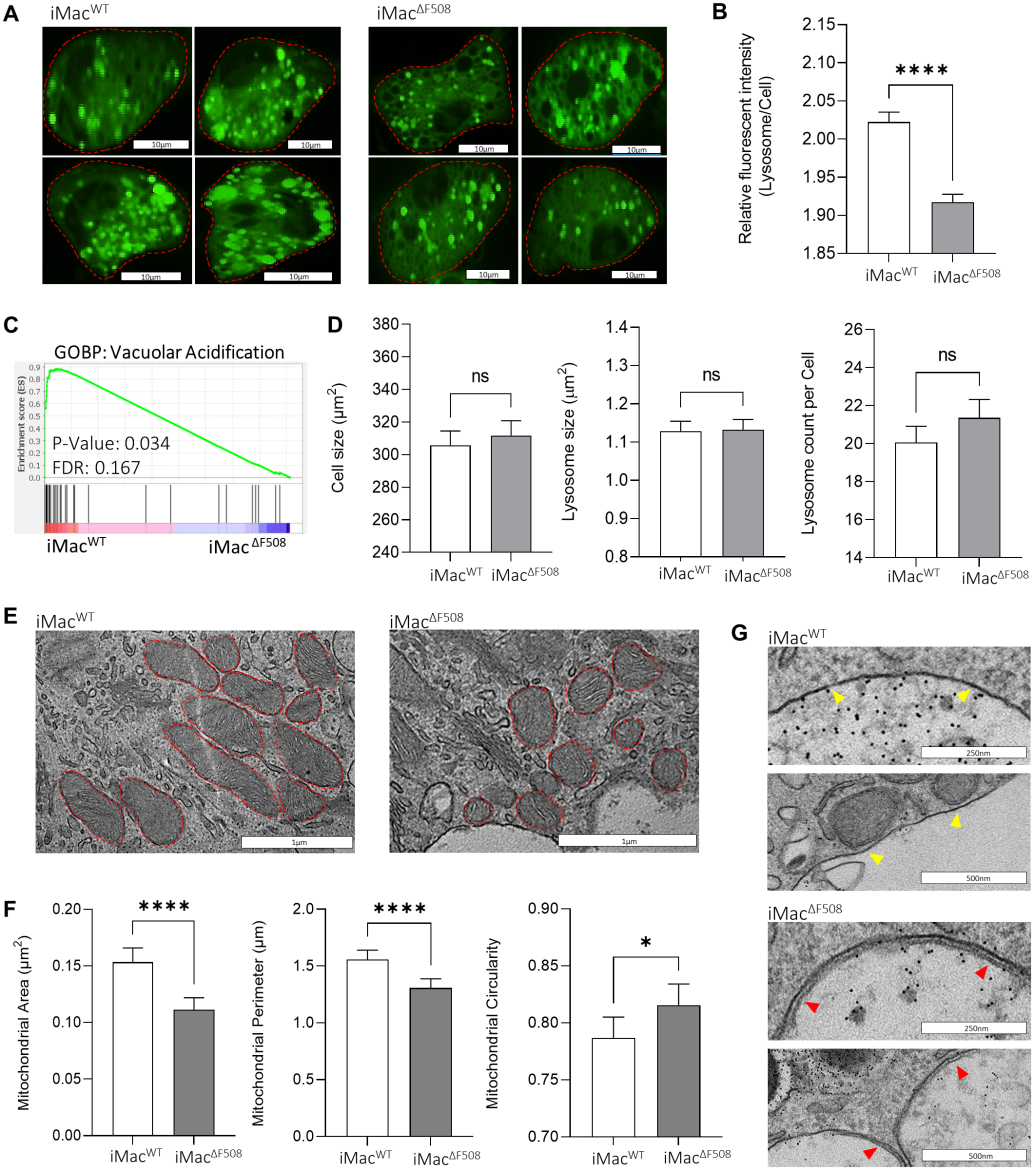
Analysis of differential organelle phenotypes between iMac^WT^ and iMac^ΔF508^ **(A)** Representative images of iMac^WT^ and iMac^ΔF508^ stained with LysoSensor Green DND189. Scale bar: 10µm. **(B)** Quantification of relative fluorescence intensity of detected acidic organelles was normalized to the background intensity of the cell and plotted for both iMac lines. Mann-Whitney-U test, bars represent mean intensity ± 95% confidence interval of analysed acidic organelles in iMac^WT^ (N=5,870) and iMac^ΔF508^ (N=6,207). **(C)** Gene set enrichment analysis comparing iMac^WT^ and iMac^ΔF508^ regarding the gene ontology biological process (GOBP) “vacuolar acidification”. **(D)** Comparison of iMac^WT^ and iMac^ΔF508^ regarding cell size, lysosome size and number of lysosomes per cell. Mann-Whitney-U test was performed on WT (N=285) and CF (N=286)). **(E)** Representative micrographs depicting mitochondria (red lines) in iMac^WT^ and iMac^ΔF508^. **(F)** Comparison of area, perimeter and circularity of mitochondria in iMac^WT^ (N=209) and iMac^ΔF508^ (N=203). Mann-Whitney-U test. **(G)** Representative images depicting the presence of double membrane vesicles in iMac^ΔF508^ (red arrows), and single membrane vesicles in iMac^WT^ (yellow arrows). Scale bar: 250 or 500µm. P-value indicated with asterisks (*p < 0.05 and ****p < 0.0001).

In order to dissect the observed dysfunctional killing capacity of iMac^ΔF508^, a relative lysosomal pH comparison between iMac lines was performed. To do so, iMac were first stained with the pH-sensitive dye Lysosensor Green DND189 which fluorescence signal increases as the environment gets more acidic. Live-cell-imaging of iMac was performed with a fluorescence confocal microscope (**Figure 3A**). For the analysis of microscopic images, a KNIME based software tool specifically programmed for this study was used, which automatically identified and measured fluorescence intensity of acidic vesicles within the cells. Thus, it was shown that iMac^ΔF508^ had a slightly but significantly less acidic lysosomal pH when compared to iMac^WT^ (**Figure 3B**). This observation was further validated by the significantly enriched transcriptome related to the biological pathway “vacuolar acidification” on iMac^WT^ when compared to iMac^ΔF508^ (**Figure 3C**). Of note, no significant difference was observed between both iMac lines regarding cell size, lysosomal size or lysosome number per cell (**Figure 3D**).

Additionally, a morphological quantitative analysis was performed regarding mitochondria with the micrographs taken of iMac^WT^ and iMac^ΔF508^ 150’ post-infection (**Figure 3E**). Mitochondria of infected iMac^ΔF508^ were significantly smaller and their shape was significantly rounder when compared to infected iMac^WT^ mitochondria (**Figure 3F**).

Lastly, an interesting observation was made from the micrographs of iMac^WT^ and iMac^ΔF508^ 150’ post-infection. The phagosomal vesicles of iMac^ΔF508^ frequently exhibit a clear double membrane, a typical feature of autophagosomes, which could not be detected on iMac^WT^ images (**Figure 3G**). Furthermore, reduced phosphorylation of the subunit p47-phox of the NADPH oxidase complex, an intrinsic enzyme of the reactive oxygen species (ROS) cascade, in iMac^ΔF508^ compared to iMac^WT^ 30min post infection points towards an impaired or defective ability of diseased iMacs to efficiently produce ROS (**Supplementary Figures 2A, B**).

### Transcriptome data highlights the hyperinflammatory phenotype of iMac^ΔF508^

In an attempt to further understand the functional differences between iMac^WT^ and iMac^ΔF508^, a transcriptomic analysis was performed. Following the previously presented intracellular bactericidal protocol, duplicate RNA isolates of both iMac lines were prepared at different time points along a *P. aeruginosa* infection (non-infected, 60’, 150’, and 450’ post-infection) and subsequently sequenced.

**Figure 4:**
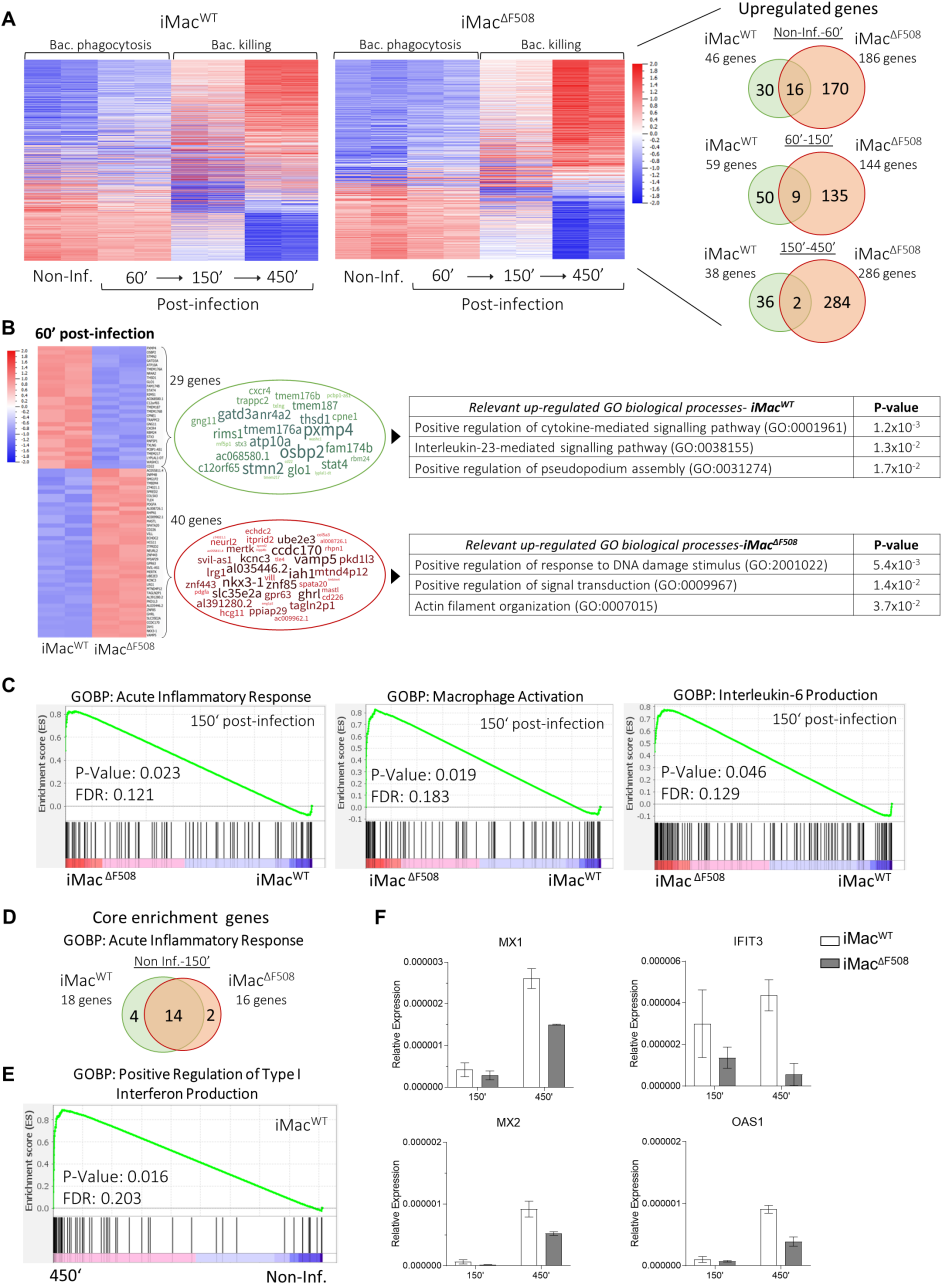
Comparative RNA expression analysis between iMac^WT^ and iMac^ΔF508^ during a *P. aeruginosa* infection (N=2 for each cell line and time point). **(A)** Heat maps (P-values < 0.01 and fold change > 2) and Venn-diagrams of differentially expressed genes in iMac^WT^ and iMac^ΔF508^ along different time points of an infection course. **(B)** Heat maps summarizing differentially expressed genes (P-values < 0.01 and fold change > 2) between iMac^WT^ and iMac^ΔF508^ 60’ post-infection (69 genes) representing relevant biological processes associated with the upregulated genes of each iMac line. **(C)** Gene set enrichment analysis comparing iMac^WT^ and iMac^ΔF508^ 150’ post-infection regarding the GOBP “acute inflammatory response”, “macrophage activation” and “Interleukin-6 production”, which were significantly upregulated in iMac^ΔF508^. **(D)** Venn-diagram of significantly upregulated core genes of the biological process “acute inflammatory response” between non-infected and 150’ post-infection time points in iMac^WT^ and iMac^ΔF508^. **(E)** Gene set enrichment analysis comparing the GOBP “positive regulation of type I interferon production” between non-infected and 450’ post-infection time points, which was significantly upregulated only on iMac^WT^. **(F)** Relative gene expression of *MX1*, *MX2*, *IFIT3* and *OAS1* genes of both iMac lines at infection times 150’ and 450’.

When looking at the significantly differential gene expression along the infection progression on each iMac line, it was evident that both, iMac^WT^ and iMac^ΔF508^, responded to the bacterial pathogen by modifying their transcriptome. However, it could be noted that iMac^ΔF508^ significantly upregulated a greater number of genes in response to the infection in comparison to iMac^WT^ (**Figure 4A**). When closely analyzing the upregulated genes, it was obvious that iMac^WT^ first upregulated biological processes related to phagocytosis, and at later stages, genes related to efficient response against infection. In contrast, iMac^ΔF508^ tended to upregulate genes fully committed to enhance cytokine response (**Supplementary Figure 3**).

The differentially expressed genes of iMac^WT^ and iMac^ΔF508^ at the same time points along the course of a *P. aeruginosa* infection are represented by heat maps (**Figure 4B**; **Supplementary Figure 4A**). Thus, it is possible to visualize that both iMac lines showed significant differential expression of 89 genes already before *P. aeruginosa* infection, which were associated with biological processes such as vascular acidification, macrophage migration and proteasome assembly. The amount of significantly differentially expressed genes got slightly reduced at earlier stages of the infection (69 genes; 60’ post-infection), when both lines showed upregulated biological processes related to the phagocytosis processes such as pseudopodium assembly and actin filament organization. However, a potentially crucial biological process was identified in iMac^ΔF508^: “Positive regulation of response to DNA damage stimulus” (**Figure 4B**). Interestingly, in the following time point, 150’ post-infection, iMac^ΔF508^ showed the upregulation of the process “Negative regulation of DNA damage” and in the last point 450’ post-infection upregulated processes of this iMac line were aimed towards efficient clearing of bacterial infection such as increasing ROS production (**Supplementary Figure 4A**).

Furthermore, a direct comparison between the lines at time point 150’ post-infection evidenced the enrichment on biological processed such as acute inflammatory response, macrophage activation and interleukin-6 production on iMac^ΔF508^, highlighting their hyperinflammatory response against the *P. aeruginosa* infection (**Figure 4C**). Despite this observation, both iMac lines significantly upregulated genes related to an acute inflammatory response from the non-infected state to 150’ post-infection (**Supplementary Figure 4B**) and as a matter of fact, the core enriched genes of this process along this time frame were mainly the same in both iMac lines (**Figure 4D**).

When the entire infection process (non-infected until 450’ post-infection) is taken into consideration, only iMac^WT^ significantly upregulated the type I interferon production (**Figure 4E**). This finding is further supported by gene expression analysis, where central genes involved in the response to type I interferon, namely *MX1*, *MX2*, *IFIT3* and *OAS1*, show a decreased expression on iMac^ΔF508^ when compared to iMac^WT^ (**Figure 4F**; **Supplementary Figure 5**).

## Discussion

In the present study, we used macrophages derived from iPSCs, originally generated from a CF p.Phe508del individual and a healthy donor (31, 32) to gain a deeper understanding of the innate immunity response to bacterial infections. In that regard, we have shown that iMac^ΔF508^ and iMac^WT^ could be successfully generated from their respective iPSC lines following an already stablished differentiation protocol (40). However, even though both iMacs lines exhibited a similar typical macrophage morphology and surface marker expression, their response to *P. aeruginosa* infection was significantly different. Of note, iMac^ΔF508^ showed a delay in the process of intracellular *P. aeruginosa* degradation of approximately 1h when compared with iMac^WT^. This delay might play a role in the decreased overall bacterial clearance capacity of diseased macrophages. Previous studies already highlighted an impaired bactericidal capacity of CF macrophages, associating this dysfunction with different cellular processes, including phagocytosis, phagolysosome formation, lysosomal acidification or ROS generation (22–25).

Despite the fact that the expression of CFTR in human macrophages is hundreds of times lower than in epithelial cells (41), functional CFTR was confirmed in macrophages in multiple studies (42–44). Furthermore, CFTR was also detected in tissue resident alveolar macrophages in minimal mRNA expression levels (45, 46) and with a phagosome/lysosome location (44). It was initially believed that the absence of functional CFTR expression in CF macrophages is linked to deficient bactericidal processes due to reduced lysosomal acidification (45), this theory is now considered to be incomplete (47). Direct acidification of the phagosome is not the primary bactericidal mechanism of pro-inflammatory macrophages, which are present in an environment such a CF lung during a bacterial infection. Instead, these macrophages mostly rely on ROS production to rapidly eliminate potential threats (48). An alternative hypothesis links the absence of functional CFTR to deficient ROS generation mechanisms in CF macrophages, as supported by our result revealing reduced phosphorylation of p47-phox in iMac^ΔF508,^ especially notable 30 minutes after *Pseudomonas aeruginosa* infection. CF macrophages have secretory lysosomes containing a less acidic lumen, which has a detrimental effect on the enzymatic synthesis of lipid raft components, leading to a lower presence of NADPH oxidase on the cell membrane and decreased ROS concentration in the *P. aeruginosa* containing endosome (44). Regardless of the specific mechanistic link between CFTR expression and macrophage bactericidal impairment, our data support the presence of less acidic organelles within iMac^ΔF508^, substantiating multiple publications, which directly or indirectly associate a decreased lysosomal acidity with a decreased killing capacity of CF macrophages (44, 45, 49). Furthermore, additional observations of organelles within macrophages 150’ post-infection, showed potential mitochondrial fragmentation in infected iMac^ΔF508^, which supports previously published data describing dysfunctional metabolism in CF macrophages, evidenced as decreased area and increased circularity of mitochondria only after bacterial infection (24). Interestingly, it has been reported that induction of autophagy by intracellular *P. aeruginosa* constitutes a mechanism used by this pathogen to escape intracellular killing (50). A typical feature of autophagolysosomes is their double membrane (51). Therefore, our observation that iMac^ΔF508^ presented vesicles formed by double membranes could constitute a hint of CF macrophages being more sensitive to this bacterial strategy to avoid degradation.

Considering the transcriptomic changes associated with a *P. aeruginosa* infection, we observed that although both cell lines responded in a largely similar manner by modifying their gene expression, certain distinct differences could be identified. iMac^ΔF508^ were transcriptomically more reactive at every single time point of the infection compared to iMac^WT^, potentially indicating a hyper-responsiveness of CF macrophages to the microbial stimulus. Interestingly, 60’ post-infection, iMac^ΔF508^ exhibited a positive regulation of response to DNA damage stimulus, probably triggered by *P. aeruginosa* as a mechanism to evade degradation, which might constitute yet another factor explaining the delayed bactericidal capacity (52). However, although both lines significantly upregulated genes related to the acute inflammatory response in the first 150’ post-infection, a side by side comparison at that time point suggest that CF macrophages exhibit a more hyper-inflammatory phenotype in comparison to WT cells, a fact already reported by others (25, 27, 46, 53). Finally, it was also noticed that iMac^ΔF508^ showed a significantly reduced Type I interferon pathway upregulation. This pathway is not only essential to orchestrate immune response against viral particles, but also in innate immunity response against threats of bacterial origin. In accordance with our data, it has been recently reported that a dampened upregulation of Type I interferon pathway is characteristic of CF macrophages infected with *P. aeruginosa* (54).

Overall, it is evident that the absence of functional CFTR in macrophages acts as a regulator influencing many biological processes associated with the bacterial clearance and effective infection resolution. Therefore, it can be hypothesized that the adoptive transfer of WT macrophages into the CF lung could assist in the prevention and resolution of bacterial infections, opening the door to a future cell therapy for pwCF, which we are currently testing in a CF murine model. The study presented here has some limitations as CF and non-CF iPSC were derived from different donors. In future research it should be considered if WT iPSC lines could be directly derived from the biallelic genetic editing of a CF iPSC line that reverts the CF causing mutation to eliminate potential donor-specific fluctuations in the generated iMac. The main advantages of iMac as a cell source for research are their reproducibility and on-demand production. This could not only facilitate the study of macrophages expressing the most common pathogenic *CFTR* variants, but also rare forms of CF allele combinations where direct access to pwCF cells would be challenging. Furthermore, once iPSC lines are generated, they can be stored, expanded, and differentiated whenever needed, eliminating the need to request further biomaterial samples from pwCF. A biobank containing iPSC cell lines generated from pwCF with different *CFTR* variant combinations could be shared between research groups around the world and used to investigate open basic research questions or even perform drug discovery trials to improve CF macrophage bactericidal functionality.

## Data Availability

The RNA sequencing data presented in the study is deposited in the GEO repository, accession number GSE276816.
