# Supplementary figures and images for: Human CFTR deficient iPSC-macrophages reveal impaired functional and transcriptomic response upon Pseudomonas aeruginosa infection
Source: Front Immunol. 2024 Nov 13;15:1397886. doi: 10.3389/fimmu.2024.1397886 (PMC11601075; doi:10.3389/fimmu.2024.1397886)

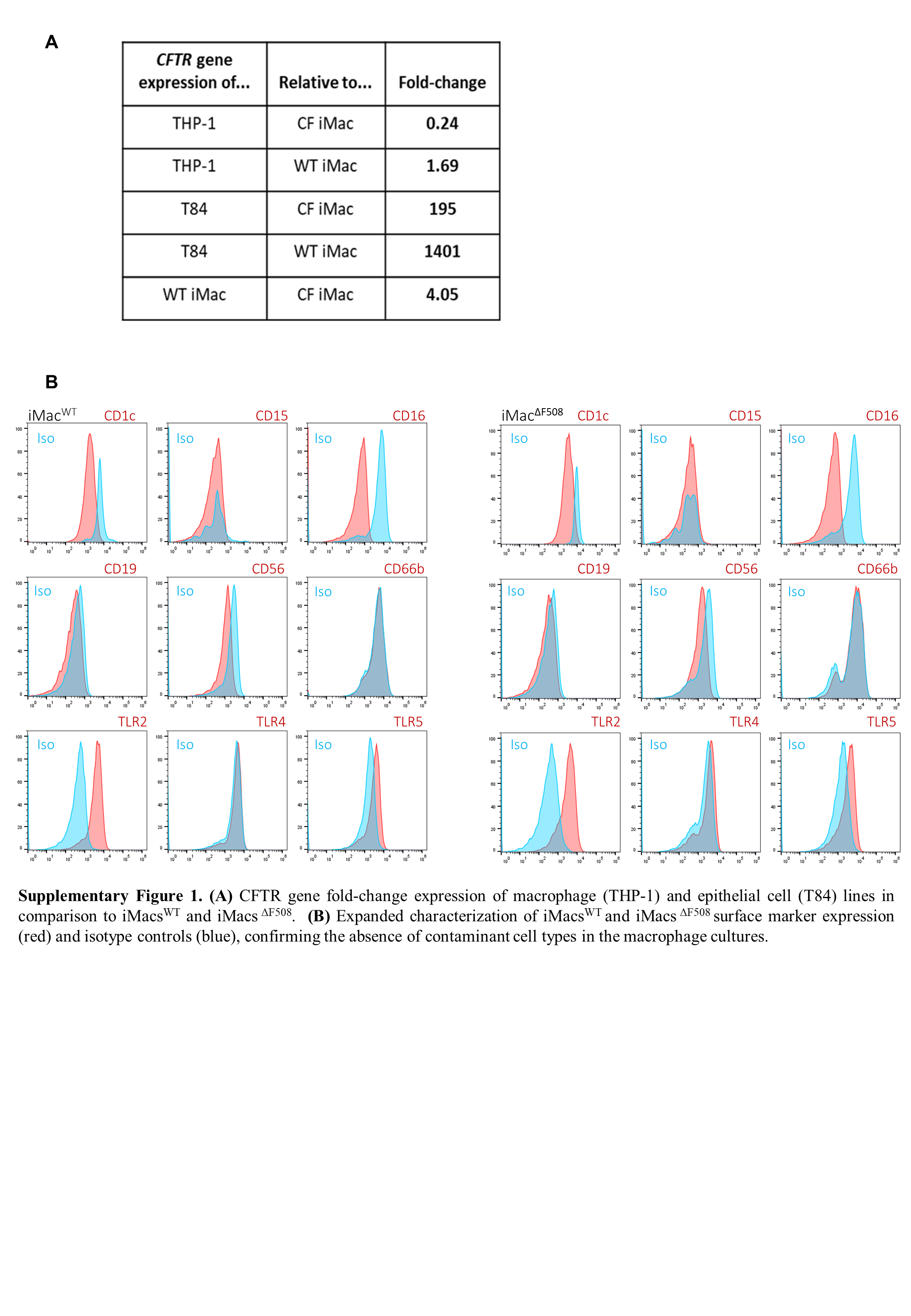

Supplement: Supplementary file 1 [file Image1.tif]

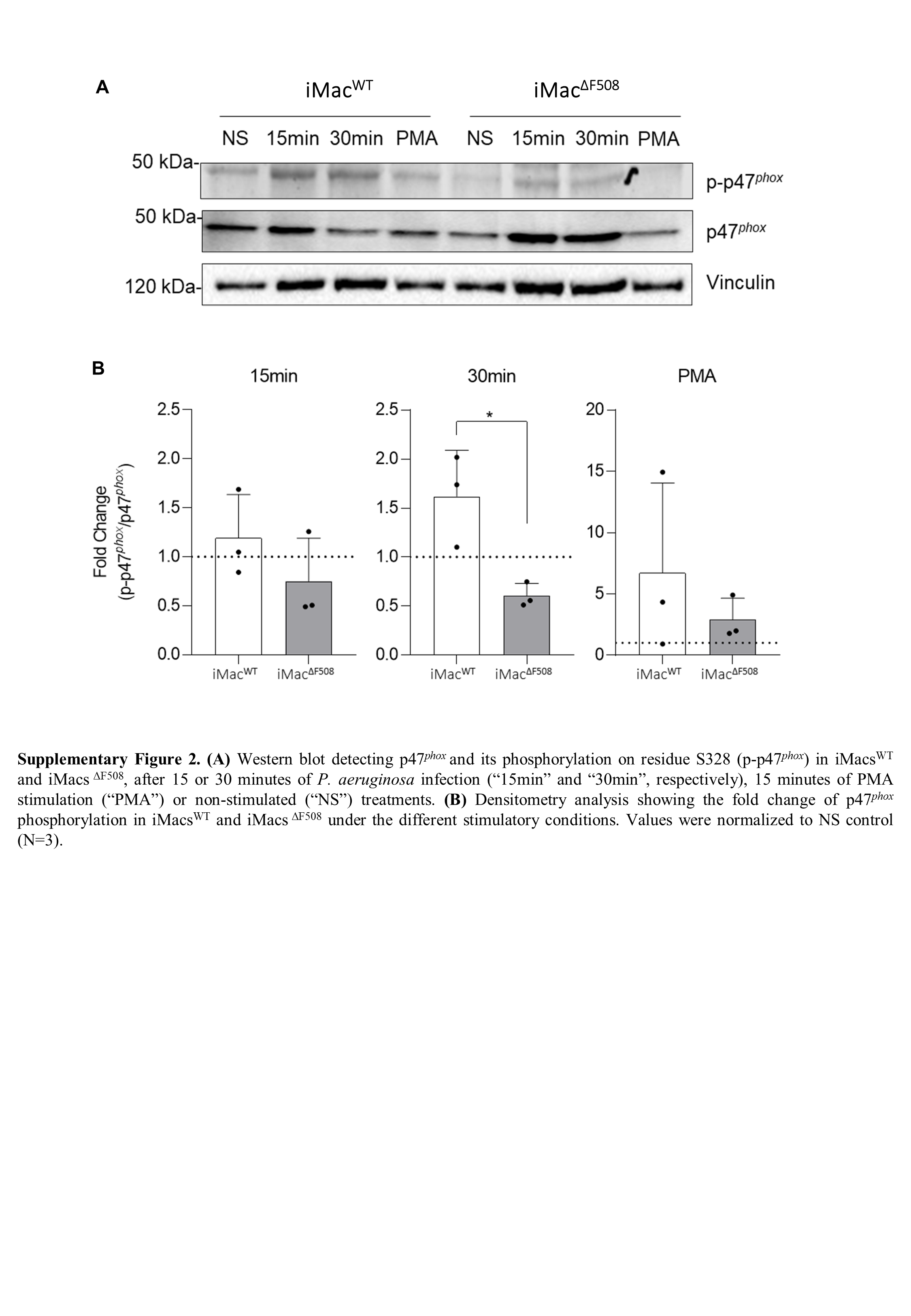

Supplement: Supplementary file 2 [file Image2.tif]

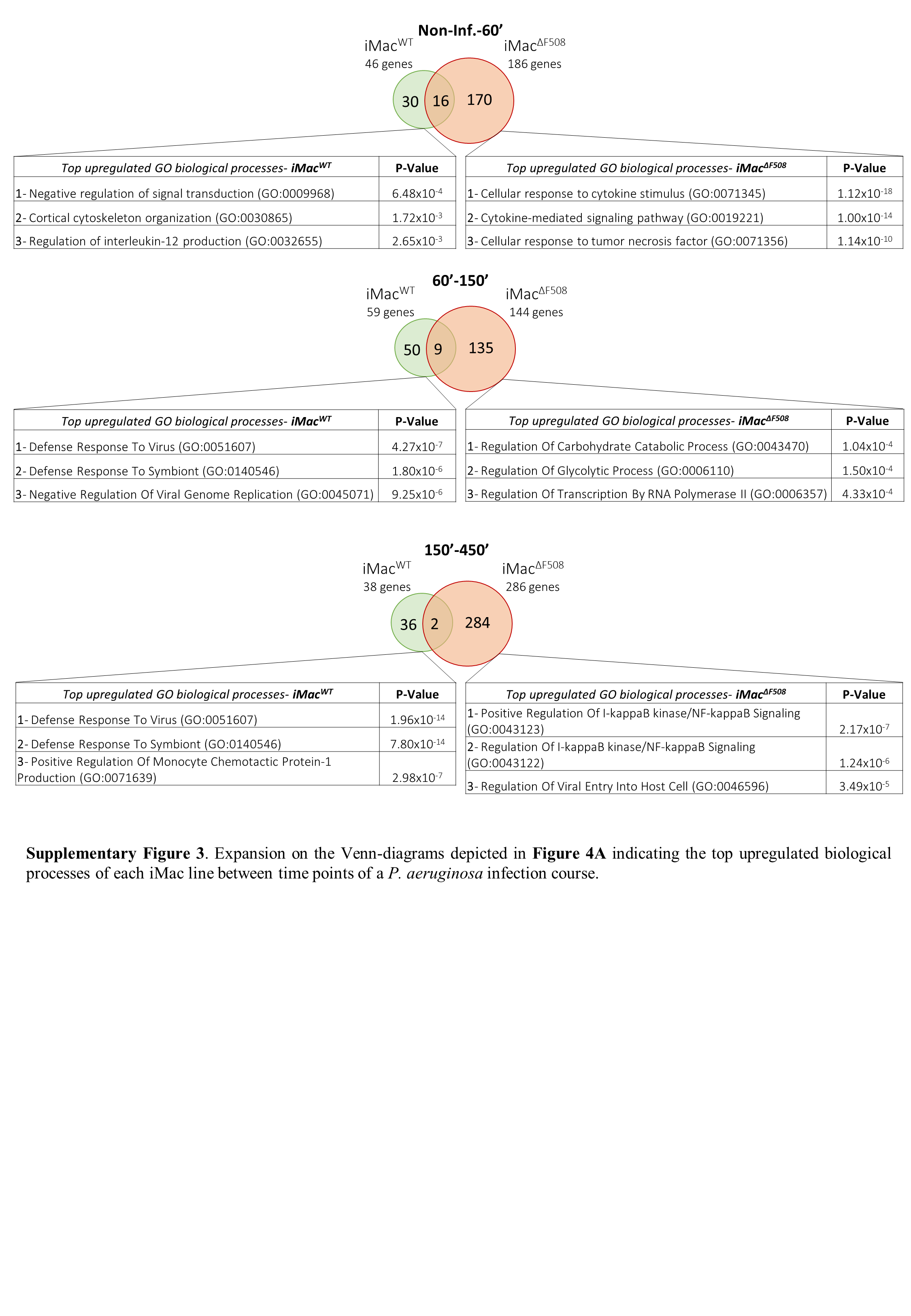

Supplement: Supplementary file 3 [file Image3.tif]

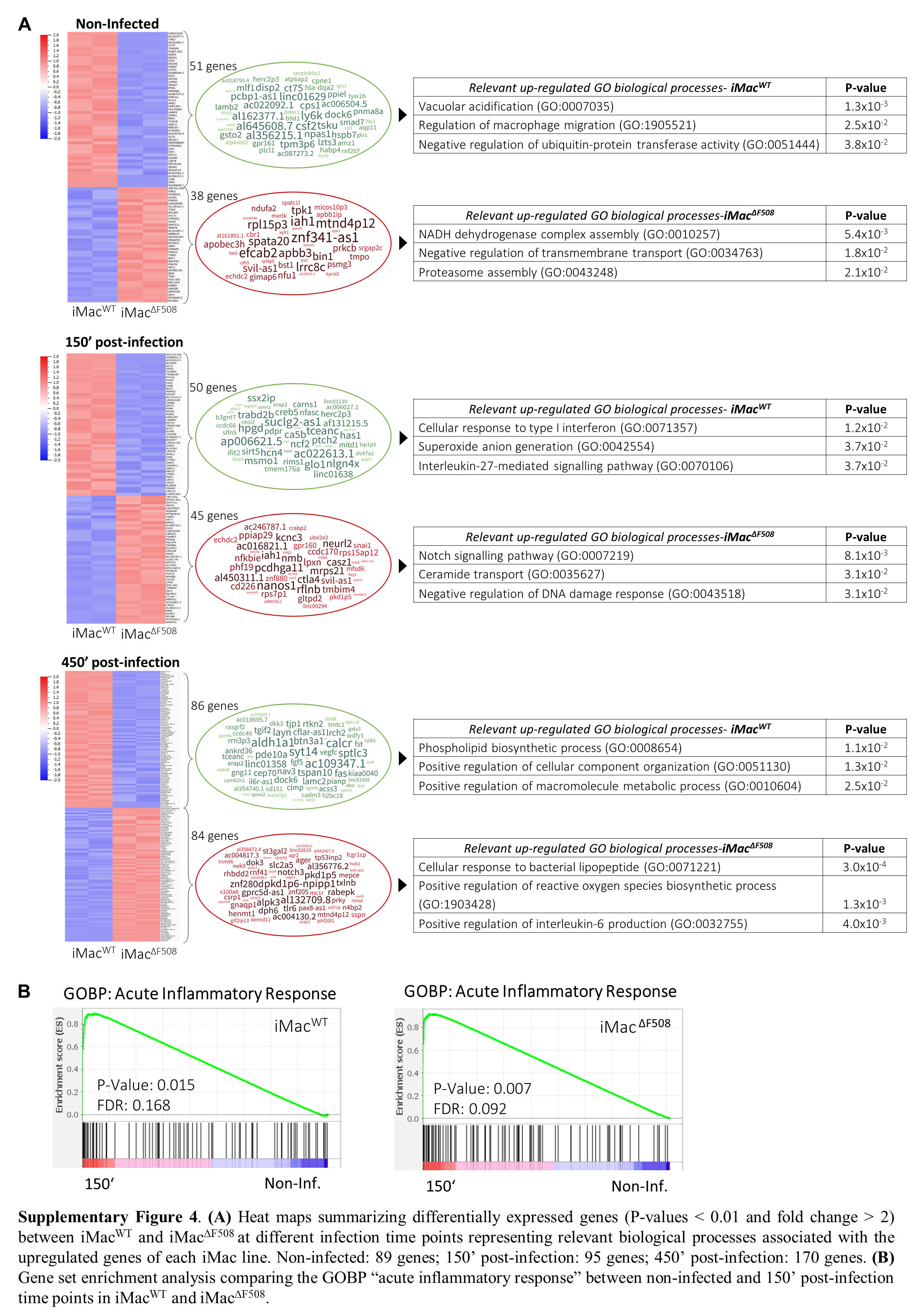

Supplement: Supplementary file 4 [file Image4.tif]

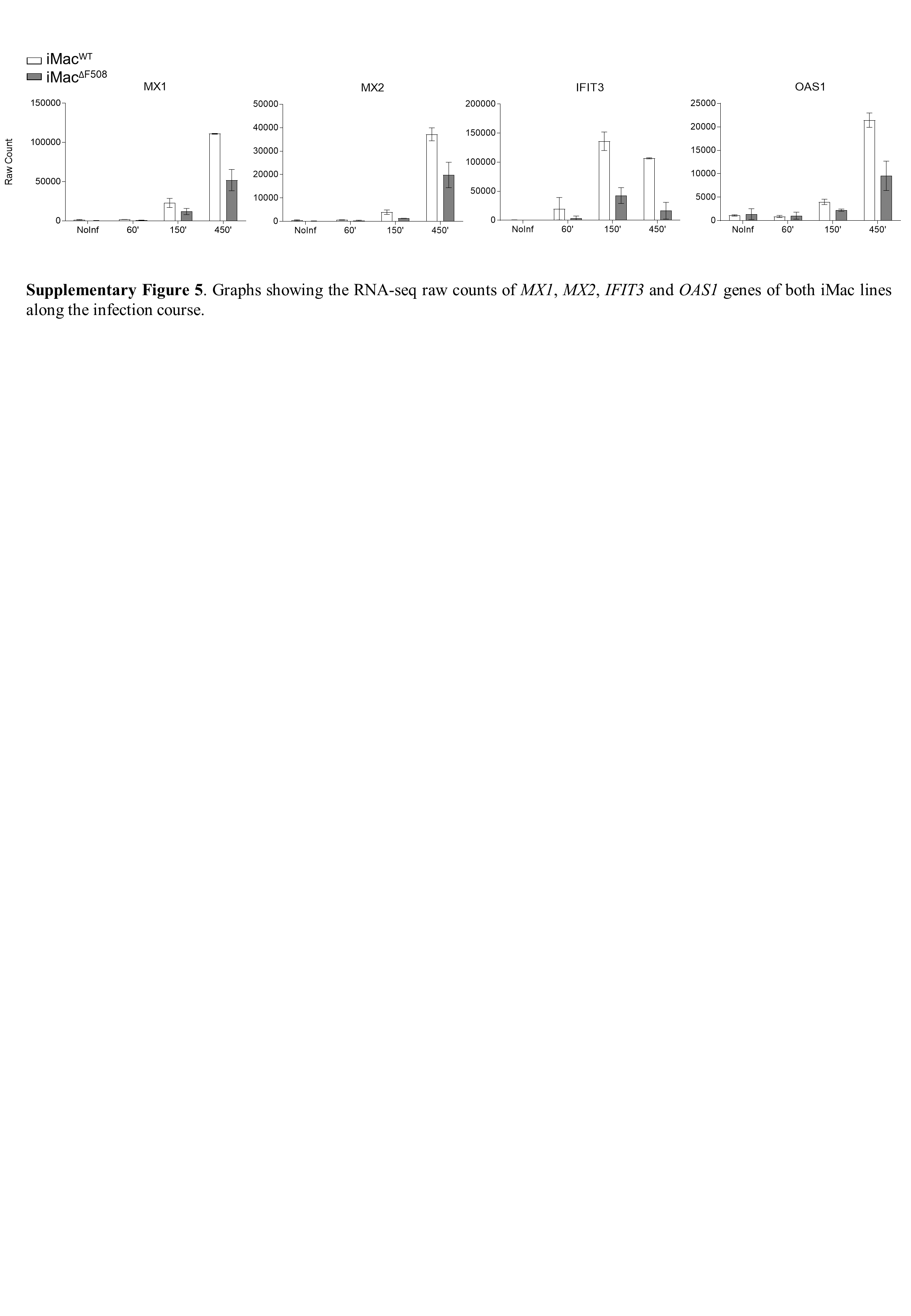

Supplement: Supplementary file 5 [file Image5.tif]
